# Epinephrine for Anaphylaxis: Underutilized and Unavailable

**DOI:** 10.5811/westjem.2015.3.25337

**Published:** 2015-04-06

**Authors:** Larissa S. Dudley, Madonna I. Mansour, Mark A. Merlin

**Affiliations:** * Newark Beth Israel Medical Center, Department of Emergency Medicine, Newark, New Jersey; † New York College of Osteopathic Medicine, Old Westbury, New York; ‡ Rutgers School of Public Health/Medical School, Piscataway, New Jersey

## INTRODUCTION

Anaphylaxis is a rapidly progressing, potentially life threatening allergic reaction that has been increasing in prevalence, most commonly triggered by foods, medications, and insect stings. Allergies in children are increasingly more common. Unfortunately, anaphylactic reactions are under-recognized, due to overlooked or under-appreciated symptoms, and therefore under-treated with epinephrine.^1^ For several years, epinephrine has been established as the drug of choice for anaphylaxis.^2^ Even a few minutes delay in the recognition and treatment of anaphylaxis can lead to hypoxia or death. Therefore, healthcare professionals and laypeople alike should be able to recognize the signs and symptoms of anaphylaxis and have accessible resources to initiate treatment.

Broadened awareness of the need for emergent anaphylactic treatment with readily available epinephrine auto-injectors, analogous to the common awareness and use of publicly housed automated external defibrillators (AEDs) in cardiac arrest, may decrease the morbidity and mortality of this rapidly progressing disorder. In 2006, Lieberman et al. reviewed articles since 1968 regarding epidemiological studies of anaphylaxis, finding approximately 50–2,000 episodes per 100,000 people with the largest incidence among children and adolescents;^3^ mortality rates approximate 0.65 to 2%.^4^ Boyce et al. found anaphylaxis accounted for 1 to 70 per 100,000 hospitalizations or emergency department visits.^5^ In 2014, Ma et al. demonstrated the annual number of hospitalizations related to anaphylaxis increased from 5,700 to 7,700 from 1999 to 2009, and from 2006 to 2009 anaphylaxis related emergency department visits and hospitalizations increased from 25,000 to 30,000 annually.^6^ The most recent figures, published in 2014, estimate the prevalence of anaphylaxis in the general population to be at least 1.6%, although probably higher.^7^ While literature strongly suggests the need for available epinephrine in schools to treat anaphylaxis,^8^ often triggered by foods, it seems reasonable and logical to have epinephrine auto-injectors available in populated public areas, similar to those where AEDs are available, for the life-saving treatment of anaphylactic reactions triggered by any allergen.

## TREATMENT AND CONSEQUENCES OF DELAYED TREATMENT

Anaphylaxis is a clinical diagnosis with symptoms that occur along a continuum. Symptoms may be as mild as itching of the eyes, nose, or skin although urticaria and tongue swelling manifest most commonly. Symptoms may progress rapidly to cardiovascular or respiratory collapse.^2^ According to The 2013 World Allergy Organization (WAO) Anaphylaxis Guidelines, clinical criteria for diagnosing anaphylaxis include any one of the following three: 1. Acute onset of an illness (minutes to several hours) with involvement of the skin, mucosal tissue, or both (e.g. generalized hives, pruritus or flushing, swollen lips-tongue-uvula) with either respiratory compromise and/or reduced blood pressure or associated symptoms of end-organ dysfunction; 2. Two or more of the following that occur rapidly after exposure to a likely allergen for that patient over minutes to several hours: a. Involvement of the skin/mucosal tissue, b. Respiratory compromise, c. Reduced blood pressure or associated symptoms, or d. Persistent gastrointestinal symptoms (e.g., crampy abdominal pain, vomiting); or 3. Reduced blood pressure after exposure to known allergen for that patient.^9^ According to the WAO 2013 Update, anaphylaxis in children is most often triggered by foods that cause respiratory symptoms, while anaphylaxis in the elderly manifests with cardiovascular symptoms, most often triggered by medications or insect stings.^3^

Intramuscular (IM) epinephrine has been well established as the first line treatment for anaphylactic reactions and should be administered immediately upon clinical suspicion. Epinephrine should be given at 0.01mg/kg, up to 0.5mg IM, typically in the lateral thigh. A repeat dose can be administered in five minutes if rapid improvement is not seen. Commercially available auto-injectors are dosed at 0.3mg for adults and 0.15mg for children.

There are no absolute contraindications for epinephrine administration to treat anaphylaxis, although the National Electronic Injury Surveillance System, which reviewed 2,333 visits for anaphylaxis during 2002, found that only 19% of patients who needed epinephrine received the medication appropriately.^11^ It is known that an inadequate dose increases the risk of a biphasic reaction,^15^ while delayed or lack of treatment can lead to hypoxia and or death.

## ADJUNCT THERAPIES

The necessity of prompt epinephrine administration cannot be stressed enough. While therapies exist to alleviate mild symptoms of allergies, let it be clear that epinephrine is the primary treatment for anaphylaxis given that no other pharmacotherapy will treat the vasodilation and bronchoconstriction characteristics of the illness. Unfortunately, antihistamine use is the most common reason providers report for not using epinephrine, leaving patients at increased risk for life threatening sequelae.^16^ Literature supports that antihistamines have no effect on anaphylaxis. Antihistamine administration is optional and use should used for the symptomatic relief of pruritus and rash, understanding that this is over a mean time of 101 minutes. Also note that administration of an antihistamine may mask the cutaneous symptoms of anaphylaxis, potentially delaying treatment with epinephrine. Despite this, healthcare professionals often inappropriately rely on diphenhydramine for anaphylactic reactions. There is no evidence for the use of corticosteroids in the acute treatment of anaphylaxis. Steroids take 4–6 hours to reach maximum effectiveness, however they may be beneficial in preventing biphasic reactions when symptoms return 6–10 hours later.

## EPINEPHRINE AUTO-INJECTOR AVAILABILITY

Most community spaces, such as schools, parks, pools, and event venues do not have patient non-specific epinephrine auto-injectors available on site. Of those locations with the drug device stocked, it may be difficult or impractical to locate and the employees or staff are often unskilled in their use. In 2008, Ben-Shosham et al. demonstrated that 48% of children prescribed an epinephrine auto injector did not have the device available at school and of those with the medication on site, 78% of the auto-injectors were kept in the office of the nurse or another administrator.^12^ Additionally, many students with food allergies do not routinely carry epinephrine. All fifty states allow epinephrine to be carried in emergency vehicles, but only seventeen states require that epinephrine be carried by all levels of emergency medical system (EMS) providers.^14^ Since many patients who have been prescribed an epinephrine auto-injector do not regularly carry one and not all basic life support ambulances carry epinephrine, because of legislation issues or cost, it is essential that life-saving epinephrine auto-injectors be readily available in community spaces and public venues.

## CURRENT PRACTICES AND NEED FOR CHANGE

Current practice revolves around the physician or licensed provider prescribing the epinephrine auto-injector and educating the patient and family on the administration, storage and use. The WAO 2013 Guidelines report that patients and their caregivers are less likely to carry their epinephrine auto-injectors and competency in their use decreases over time.^10^ Ercan et al. surveyed 237 teachers and found only 10% were familiar with an epinephrine auto-injector and 4% were aware of proper administration.^13^

A study in the United Kingdom revealed the onset of anaphylaxis leading to cardiopulmonary arrest caused by food reactions averaged 25–30 minutes, 10–15 minutes for insect stings, and 10–20 minutes for drugs consumed out-of-hospital.^2^ Many healthcare professionals incorrectly treat anaphylactic reactions by administering epinephrine though alternate routes or by administering second line therapies first. In a study of 103 patients experiencing allergic reactions or anaphylaxis, 12 patients received intramuscular epinephrine before the arrival of EMS, 15 patients received epinephrine by EMS providers: 4 patients received intravenous epinephrine, and 11 patients received epinephrine subcutaneously.^17^ Given that 55% or more of people receiving epinephrine out-of-hospital had no prior severe allergies or anaphylaxis,^8^ it is reasonable to propose a model for publicly available epinephrine auto-injectors in populated community locations.

The American Heart Association has established a “chain of survival” for cardiac arrest that includes the following: 1. immediate recognition of cardiac arrest and activation of the emergency response system; 2. early cardiopulmonary resuscitation; 3. rapid defibrillation; 4. effective advanced life support; and 5. integrated post-cardiac arrest care.^18^ Iwami et al. studied public AED use in Japan and found railway stations to be the most common site for shock deliveries, likely related to population concentration. In the US, sports facilities, airports, and amusement areas are the most common places where AEDs are used.^18^,^19^ As literature supports, anaphylaxis is most commonly induced by exposure to food, medications, or stings. Therefore it seems only reasonable to have auto-injectors available in the same community areas as those where AEDs are stocked. Since 13–65% of anaphylaxis cases are thought to be food related,^5^ patient non-specific epinephrine auto-injectors ought to also be available and accessible in schools, cafeterias, malls, and places where food is served. The 2014 study by Murakami et al. found that increasing publicly available AEDs decreased the average time from collapse to defibrillation to five minutes.^19^ Similar patterns would likely be seen if epinephrine auto-injectors were readily available in public locations.

Reasonably, concerns regarding cost, safety, and education will arise. While this proposal is not recommending any one brand of auto-injector, Mylan Specialty L.P., the distributor and marketer of EpiPen and EpiPen Jr. Auto-Injectors, created the EpiPen4Schools program where eligible schools can receive up to four auto-injectors and purchase discounted auto-injectors, two per package, for $112.^20^ Muck et al., in their retrospective cohort study, reviewed data over a six year period on patients reported to six poison control centers after accidental epinephrine auto-injector injections. 365 cases were reported; most cases were treated supportively under observation, 29 required mild vasodilatory therapy, and all were discharged home.^21^

## CONCLUSION

The literature widely supports that prompt administration of intramuscular epinephrine is essential in the treatment of anaphylaxis. Anaphylactic reactions are becoming increasingly common and can progress to cardiopulmonary arrest within minutes. It is essential that patient non-specific epinephrine auto-injectors be available in public locations including schools, parks, pools, airports, public venues, and shopping malls. Increasing the availability of epinephrine necessitates the need for education of healthcare professionals, first responders, and the general public on recognizing classic anaphylactic symptoms and how to properly initiate treatment with an available pre-dosed epinephrine auto-injector.
